# Dissecting the Delirium: A Case of Triple Vessel SCAD Secondary to Encephalitis

**DOI:** 10.1155/cric/4183983

**Published:** 2026-08-26

**Authors:** Katie Doyle, Patrick Savage, Paul Johnston

**Affiliations:** ^1^ Department of Cardiology, Royal Victoria Hospital, Belfast, UK, rvh.on.ca

**Keywords:** spontaneous coronary artery dissection (SCAD), triple vessel SCAD, viral encephalitis

## Abstract

Spontaneous coronary artery dissection (SCAD) is an infrequently diagnosed cause of an acute coronary syndrome (ACS). In many cases, no clear precipitant is identified. In this case, we present a novel presentation of triple vessel SCAD in a patient presenting with viral encephalitis. Despite an initial conservative strategy, haemodynamic instability and involvement of the left main stem (LMS) ultimately necessitated emergent coronary intervention with a ‘shotgun’ LMS bifurcation strategy.

## 1. Introduction

SCAD is a recognised cause of an acute coronary syndrome in 1%–4% of all ACS cases and is markedly more common in females compared with males (9:1) [1]. SCAD results from the development of a haematoma within the tunica media, which then leads to separation of the intimal layers; this can lead to compression of the true lumen causing myocardial ischaemia and/or infarction [2]. It is not causally associated with the classical ACS risk factors such as coronary arteriosclerosis and hyperlipidaemia. Instead, it is thought to be an interplay of predisposing risk factors and precipitating factors. Triggers previously well‐documented include intense physical or emotional stress, but often no trigger or stressor is identified. Multiple previous studies have identified common underlying risk factors such as female sex, pregnancy, fibromuscular dysplasia and underlying systemic inflammatory conditions (systemic lupus erythematosus, rheumatoid arthritis and Crohn′s disease) [3]. More recently, associations with COVID‐19 infection have also been documented and are theoretically thought to occur due to an inflammatory response leading to increased fragility of coronary vessels [4]. SCAD presents several diagnostic challenges and indeed in over half of presenting cases, an underlying cause is never found. Furthermore, many cases go undiagnosed. We present a novel case where viral encephalitis precipitated triple vessel SCAD ultimately requiring percutaneous coronary intervention.

## 2. Case Presentation

In this case report, a 61‐year‐old female presented to her local district general hospital with neurological symptoms including headache, dysphasia and confusion. Initial CT imaging excluded a haemorrhagic stroke. An initial blood panel demonstrated a lymphocytosis (51.1% predominance) with a raised C‐reactive protein (CRP) of 37.5 mg/L, which prompted a lumbar puncture. Cerebrospinal fluid (CSF) demonstrated an elevated protein of 1.48 g/L, normal glucose of 3.81 mmol/L and elevated white cell count (WCC) of 94 cells/mm^3^, which were 100% lymphocytes. Both viral PCR and culture returned negative. However, the raised protein and lymphocytic pleocytosis raised clinical suspicion of encephalitis. Following neurology review, she was commenced on empiric treatment with intravenous acyclovir and pulsed methylprednisolone. Agitation and confusion made obtaining brain magnetic resonance imaging (MRI) difficult, and subsequent images were degraded by movement artefact, limiting its diagnostic utility. MRI was repeated 10 days later when she had clinically improved and identified no abnormal enhancement.

**Figure 1:**
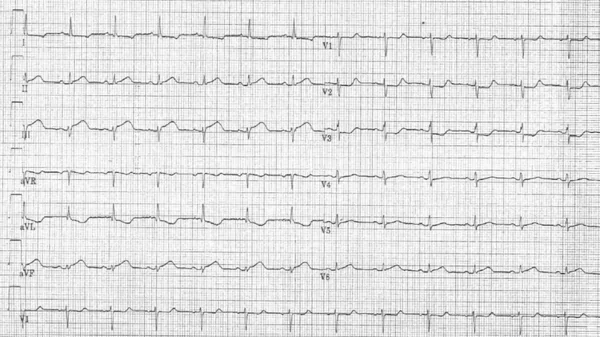
ECG demonstrating inferior ST elevation with reciprocal ST depression in keeping with a posterior myocardial infarction.

**Figure 2:**
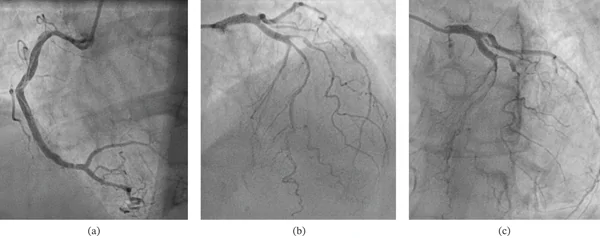
Initial coronary angiography demonstrating a longitudinal radiolucent filling defect in a spiral pattern in keeping with a Type 1 dissection in the (a) right coronary artery and diffuse long tubular stenosis affecting both the (b) LAD and (c) LCX in a Type 2b pattern.

Ten days into her admission, her agitation and confusion were improving when she developed severe chest pain with significant electrocardiogram (ECG) changes in keeping with a posterior myocardial infarction (Figure 1). She was transferred to the Royal Victoria Hospital for urgent angiography. This demonstrated a radiolucent flap extending in a spiral fashion down the right coronary artery in addition to several long diffuse tubular lesions extending distally in both the LAD and LCx (Figure 2). It was felt that these lesions represented Type 1 SCAD in the RCA and Type 2b in the left coronary system. The differential for SCAD includes atherosclerosis, spasm and thromboembolism; however, in this case, the overlap of predisposing factors, mode of presentation in addition to the pattern of distribution of disease noted, in our opinion, supported a diagnosis of SCAD as has been described in other case reports [5, 6].

Given that she was haemodynamically stable with TIMI III flow, a conservative management strategy was initially adopted. In light of the possibility of an underlying arteriopathy, cross‐sectional imaging was arranged to rule out any evolving cerebral or aortic pathology, both of which were normal.

Further local neurology input was sought at this time and interestingly, a subsequent electroencephalogram (EEG) demonstrated diffuse slowing (1.2–2.8 Hz) and generalised bifrontal single sharp waves of duration 142–185 ms of amplitude up to 234 uV, which were felt to be in keeping with encephalitis. Further screening to exclude autoimmune or paraneoplastic precipitants was negative. Additionally, infectious diseases were consulted to rule out esoteric aetiology, such as Q fever and Lyme disease; however, tests for both were ultimately negative. Indeed, in the intervening period, empirical treatment for viral encephalitis proved effective, with substantial improvements in the patient′s cognitive function noted over the following days and CSF WCC improving to 15 cells/mm^3^.

**Figure 3:**
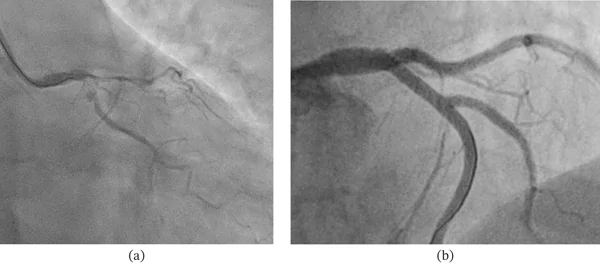
(a) Coronary angiography demonstrating flow limiting dissection of the left main stem. (b) Angiography postbifurcation PCI to LMS, LAD and LCx, utilising a simultaneous kissing stent ‘shotgun’ technique.

Unfortunately, 4 days after her index angiogram, she developed further chest pain with inferior ST segment elevation which prompted urgent repeat angiography. Inflammatory markers remained elevated at this stage with a WCC of 13.2 × 10∗9/L and CRP of 40.2 mg/L. Her RCA was now occluded, which was wired and ultimately treated successfully with three overlapping drug eluting stents (File S1). Following a brief return to the ward, further haemodynamic instability and agitation prompted re‐evaluation of the left coronary system, which was now found to have flow limiting dissections affecting all main vessels (File S2). The presence of flow limiting dissections extending to her left main stem posed significant jeopardy and thus simultaneous kissing stents were deployed from both the left anterior descending and circumflex artery back to the left main (Figure 3). Treatment of her diagonal was also required utilising a T and crush technique. Following a period of convalescence on the ward she was fit for discharge with a resolution in her neurological impairment. After nearly 10 years of follow‐up, the patient has remained free of any cardiac events.

Despite extensive investigation, a unifying diagnosis such as an inherited arteriopathy, immunologic process, or an infective precipitant was not identified. The initial diagnosis of viral encephalitis is presumed to be the most probable and indeed reflects a rare and otherwise undocumented cause of SCAD.

## 3. Discussion

There is an element of diagnostic ambiguity in this case given the inconclusive MRI head findings. A presumed diagnosis of viral encephalitis was made on the basis of several positive findings including clinical presentation, CSF obtained with raised protein and WCC and EEG findings. Furthermore, an array of tests to exclude various other pathologies were noted to be negative. The association of viral encephalitis with SCAD has not been previously documented, and we propose this case as evidence of a possible aetiological overlap between the two conditions. However, we do acknowledge other possible contributory factors known to be precipitants of SCAD such as severe agitation, autonomic activation and catecholamine surge which may be relevant in this case. It must be noted that many cases of SCAD go undiagnosed, and in those presenting to cardiac services less than half have an identifiable cause [7]. Indeed, the prevailing hypothesis of intimal separation triggered by increased shear stress could in theory be mediated by any condition that drives elevated catecholamine production or intra‐abdominal pressure. We propose that encephalitis may indeed predispose to this condition and the diagnosis of SCAD should be considered in these patients, in particular in the context of an acute coronary syndrome presentation.

## Funding

No funding was received for this manuscript.

## Consent

Verbal consent was obtained from the patient for publication of this case report, including accompanying angiography images.

## Conflicts of Interest

The authors declare no conflicts of interest.

## Supporting Information

Additional supporting information can be found online in the Supporting Information section.

## Supporting information


**Supporting Information 1** File S1: (a) Coronary angiography of the right coronary artery demonstrating flow limiting dissection associated with inferior STE and ongoing chest pain. (b) Treatment with three overlapping 3.0/24‐, 4.0/32‐ and 4.0/38‐mm drug eluting stents placed distal to proximally, respectively.

**Supporting Information 2** File S2: Coronary angiogram demonstrating flow limiting dissections affecting the LAD/LCX back to LMS. A small injection of contrast was administered with no further injections to avoid further propagation.

## Data Availability

The data that support the findings of this study are available from the corresponding author upon reasonable request.

## References

[1] Gurgoglione F. L. , Rizzello D. , Giacalone R. , Ferretti M. , Vezzani A. , Pfleiderer B. , Pelà G. , de Panfilis C. , Cattabiani M. A. , Benatti G. , Tadonio I. , Grassi F. , Magnani G. , Noni M. , Cancellara M. , Nicolini F. , Ardissino D. , Vignali L. , Niccoli G. , and Solinas E. , Precipitating Factors in Patients With Spontaneous Coronary Artery Dissection: Clinical, Laboratoristic and Prognostic Implications, International Journal of Cardiology. (2023) 385, no. 385, 1–7, 10.1016/j.ijcard.2023.05.027. 37211051

[2] Stanojevic D. , Apostolovic S. , Kostic T. , Mitov V. , Kutlesic-Kurtovic D. , Kovacevic M. , Stanojevic J. , Milutinovic S. , and Beleslin B. , A Review of the Risk and Precipitating Factors for Spontaneous Coronary Artery Dissection, Frontiers in Cardiovascular Medicine. (2023) 10, 1273301, 10.3389/fcvm.2023.1273301, 38169687. 38169687 PMC10758453

[3] Smaardijk V. R. , Mommersteeg P. M. , Kop W. J. , Pellegrini D. , van Geuns R. J. , and Maas A. H. , Psychological and Clinical Characteristics of Patients With Spontaneous Coronary Artery Dissection: A Case-Control Study, International Journal of Cardiology. (2021) 323, no. 323, 1–6, 10.1016/j.ijcard.2020.08.045. 32798624

[4] Hayes S. N. , Tweet M. S. , Adlam D. , Kim E. S. H. , Gulati R. , Price J. E. , and Rose C. H. , Spontaneous Coronary Artery Dissection, Journal of the American College of Cardiology. (2020) 76, no. 8, 961–984, 10.1016/j.jacc.2020.05.084. 32819471

[5] Paraskevaidis S. , Theofilogiannakos E. K. , Chatzizisis Y. S. , Mantziari L. , Economou F. , Ziakas A. , Hadjimiltiades S. , and Styliadis I. H. , Spontaneous Dissection of Right Coronary Artery Manifested With Acute Myocardial Infarction, Open Cardiovascular Medicine Journal. (2010) 4, no. 1, 178–180, 10.2174/1874192401004010178, 21127744. 21127744 PMC2995159

[6] Akyuz A. , Alpsoy S. , and Akkoyun D. C. , Spontaneous Coronary Artery Dissection and Woven Coronary Artery: Three Cases and a Review of the Literature, Korean Circulation Journal. (2013) 43, no. 6, 411–415, 10.4070/kcj.2013.43.6.411, 23882291. 23882291 PMC3717425

[7] Saw J. , Aymong E. , Sedlak T. , Buller C. E. , Starovoytov A. , Ricci D. , Robinson S. , Vuurmans T. , Gao M. , Humphries K. , and Mancini G. B. J. , Spontaneous Coronary Artery Dissection: Association With Predisposing Arteriopathies and Precipitating Stressors and Cardiovascular Outcomes, Circulation: Cardiovascular Interventions. (2014) 7, no. 5, 645–655, 10.1161/CIRCINTERVENTIONS.114.001760. 25294399

